# Contribution of epithelial-mesenchymal transitions to organogenesis and cancer metastasis

**DOI:** 10.1016/j.ceb.2018.06.008

**Published:** 2018-12

**Authors:** Kyra Campbell

**Affiliations:** 1 Bateson Centre, Firth Court, University of Sheffield, Western Bank, Sheffield, UK; 2 Department of Biomedical Science, Firth Court, University of Sheffield, Western Bank, Sheffield, UK

## Abstract

The epithelial-to-mesenchymal transition (EMT) plays crucial roles during development, and inappropriate activation of EMTs are associated with tumor progression and promoting metastasis. In recent years, increasing studies have identified developmental contexts where cells undergo an EMT and transition to a partial-state, downregulating just a subset of epithelial characteristics and increasing only some mesenchymal traits, such as invasive motility. In parallel, recent studies have shown that EMTs are rarely fully activated in tumor cells, generating a diverse array of transition states. As our appreciation of the full spectrum of intermediate phenotypes and the huge diversity in underlying mechanisms grows, cross-disciplinary collaborations investigating developmental-EMTs and cancer-EMTs will be fundamental in order to achieve a full mechanistic understanding of this complex cell process.

**Current Opinion in Cell Biology** 2018, **55**:30–35

This review comes from a themed issue on **Differentiation and disease**

Edited by **Katja Röper** and **Xosé R Bustelo**

For a complete overview see the Issue and the Editorial

Available online 11th July 2018

**https://doi.org/10.1016/j.ceb.2018.06.008**

0955-0674/Crown Copyright © 2018 Published by Elsevier Ltd. This is an open access article under the CC BY license (http://creativecommons.org/licenses/by/4.0/).

## Introduction

The epithelial-to-mesenchymal transition (EMT) describes a cellular process during which epithelial cells transition to a mesenchymal cell state. A deceptively simple term, first coined to describe a cell behaviour observed by Elizabeth Hay during gastrulation (see Box 1) in vertebrate embryos [1], it has generated many heated debates over the years. Classically, EMT was thought of as a binary decision, involving the transition from a completely epithelial to a fully mesenchymal cell [2], which forms only transient contacts with its neighbours [3, 4, 5]. However, recent studies have pointed to a much more fluid transition, where cells may adopt a continuum of phenotypes between the ‘extreme’ epithelial and mesenchymal cell states (reviewed in [6, 7, 8]). Our understanding of EMT as a single program has also evolved, as we now know that there are many ways for a cell to affect an EMT. For example, the molecular mechanisms underlying developmental-EMTs varies greatly, even between different tissues within the same organism, as there is a context dependence of EMT activation with input from both cell-intrinsic and extrinsic factors. Here I will focus on key-concepts that are emerging from accumulative studies of developmental-EMTs, and how these relate to the current debate on the role of EMT in cancer.

Box 1 **Glossary**

**Epiblast**

The epiblast forms one of two distinct layers arising from the innermost cells in pre-gastrulation amniote embryos, and gives rise to the embryo proper.

**Gastrulation**

Gastrulation is the process during embryonic development that changes the embryo from a blastula with a single layer of cells to a gastrula containing multiple layers of cells. It is during this stage that the three germ layers, the ectoderm, mesoderm and endoderm are formed.

**Endoderm and mesoderm**

The mesoderm and endoderm are two of the intitial three germ cell layers (mesoderm, endoderm and ectoderm) and are formed by the process of gastrulation.

**Mesendoderm**

An embryonic tissue layer which differentiates into both endoderm and mesoderm.

**Neural crest cells**

A group of cells unique to vertebrates that arise from the embryonic ectoderm cell layer, migrate through the embryo and give rise to diverse cell lineages, including melanocytes, craniofacial cartilage and bone, smooth muscle, and peripheral and enteric neurons and glia.

**Cranial neural crest**

A subset of neural crest cells derived from the anterior-most part of the neural tube, and contribute to the development of most craniofacial structures in vertebrates.

Alt-text: Box 1

## A spectrum of EMTs occurs during development

**Figure 1:**
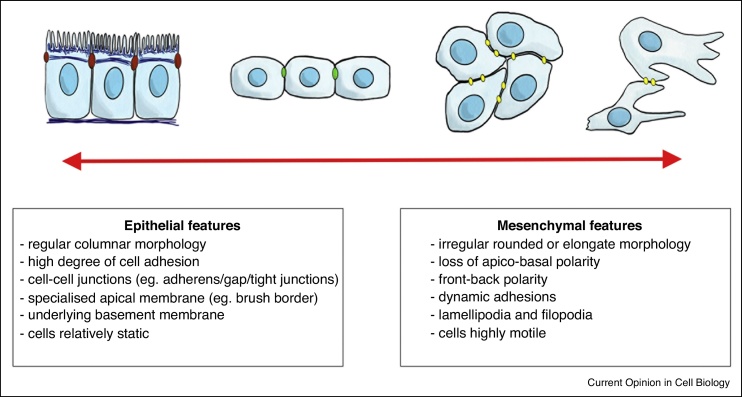
The ‘spectrum’ model for Developmental-EMTs. Almost no cell feature is unique for an epithelial, nor for a mesencyhmal cell. Instead a spectrum of cell phenotypes are seen between more differentiated epithelial and mesenchymal cell states. The accumulated loss or gain of epithelial/mesenchymal features results in a graded spectrum of cell behaviours that cells can adopt in a fluid and reversible manner. The brown junctions represent mature adherens junctions, green delineates immature junctions, and yellow show dynamic adhesions.

Considering a highly differentiated epithelial cell and an individually migrating mesenchymal cell as extremes, the accumulated loss or gain of various combinations of epithelial and mesenchymal features leads to a whole spectrum or continuum of intermediate EMT phenotypes (Figure 1). There is a great morphological variation in the initial epithelial phenotype prior to EMT (reviewed in [9]), from cells which possess fully formed junctions and an underlying basement membrane such as epiblast cells (see Box 1) in amniotes [10,11], to the primitive epithelial cells that give rise to the mesendoderm (see Box 1) in *Xenopus* and fish which possess just apico-basal polarity and immature junctions (Figure 1, [12]). A common feature of the transition to a mesenchymal state is that cells lose apico-basal polarity and stable junctions, but there is a similar continuum of mesenchymal phenotypes that result from this transition. These range from cells which migrate collectively and make cadherin based cell–cell contacts, such as *Drosophila* endoderm (see Box 1) cells and zebrafish and *Xenopus* mesoderm (see Box 1) [13,14^••^,^15^,^16^], to cells which migrate individually, and make only transient cell contacts, such as the majority of migrating neural crest (see Box 1) cells in chicks [17].

Given its potential role in cancer progression and other diseases such as fibroses, there has been great emphasis placed on defining an EMT according to the loss and gain of molecular markers. However, cells that only transition partway towards a mesenchymal state may not repress epithelial markers such as *E-Cadherin*, nor activate mesenchymal genes such as *vimentin* or *fibronectin*. In fact, there are very few features that are unique to an epithelial or a mesenchymal cell type [7,10], and cells are often found possessing a combination of so-called epithelial and mesenchymal markers [13,18^•^]. Thus, the classification of an EMT according to markers can be misleading. For example, while loss of apico-basal polarity and dissolution of junctions can occur downstream of the transcriptional repression of *E-Cadherin* (reviewed in [4]), it can also be driven by alternative mechanisms during which *E-Cadherin* remains transcriptionally active [13]. This suggests that it may be better to describe EMTs using morphological criteria, rather than molecular markers, and the tissue type, cell morphology and biological context all need to be taken into account.

## Molecular mechanisms underlying developmental EMTs

The transcriptional repression of E-Cadherin has long been considered a critical step in, and even a landmark for, EMT [3,[14^••^],^19^]. A key component of adherens junctions, E-Cadherin plays a highly conserved role in maintaining tight adherence between epithelial cells, with transcriptional downregulation of E-Cadherin pushing cells towards a mesenchymal phenotype [20,21]. However, a number of recent findings suggest that the relationship between E-Cadherin and the mesenchymal state may be more complex. First, a number of embryonic cell types such as endoderm [13,14^••^], mesendoderm [12,22] and a subset of neural crest cells, cranial neural crest (see Box 1) [23], have been found to adopt many mesenchymal features, including migration, while actively transcribing E-Cadherin. Second, while expression of so-called EMT-transcription factors such as Twist, Snail and Slug leads to a downregulation of E-Cadherin transcription in gastrulating embryos, after internalisation actively migrating cells retain E-Cadherin protein for much longer than previously thought [18^•^,^24^,^25^]. Third, E-Cadherin overexpression is not sufficient to block EMT in many cell contexts, including mesoderm [18^•^,^26^], neural crest [23,27^••^] or MDCK cells [28]. Finally, E-Cadherin has been found to play an active role in the migration of mesenchymal cells, mediating their cohesion through dynamic cell adhesion [14^••^]. Consistently, there is an increasing number of cases in which downregulation of E-Cadherin in migrating cells leads to a complete block in their migration [12,22,23,29, 30, 31, 32]. Taken together these data suggest that rather than being a marker of epithelial versus mesenchymal state, the level of E-Cadherin in a mesenchymal cell is likely to correlate with the degree to which the cells are migrating collectively or not.

Loss of apico-basal polarity and dissolution of junctions are universal morphological features of EMT, and while this can be achieved in part through transcriptional repression of E-Cadherin, many developmental systems point to alternative mechanisms. In the *Drosophila* endoderm, the GATA transcription factor Serpent drives EMT through the direct repression of the key apical polarity protein *crumbs*, which induces a loss of polarity and junctional disassembly [13]. In contrast, in the *Drosophila* mesoderm, recent studies point to an important role for posttranslational modifications of junctional proteins [33^••^,^34^]. In other organisms the underlying basement membrane needs to be broken down for EMT to take place. In gastrulating chick embryos, for example, a downregulation of basally localized RhoA activity disrupts microtubule stability, causing basement membrane breakdown and facilitating EMT [25].

Another early step driving the escape of epithelial cells from their tissue of origin are fluctuations in actomyosin contractility, which generates anisotropic increases in tension. For example, in the early *Drosophila* ectodermal epithelium neural stem cells delaminate as single cells to give rise to the nervous system. A recent study showed that cell-autonomous myosin-driven anisotropic junction loss and apical constriction drives the internalization of these cells [35]. Similarly, actomyosin contractility acts in concert with disruption of adhesions to drive delamination and EMT in chick neural crest cells, with contraction at the apical side of the cell coupled with loss of apical adhesions [36]. Anisotropic levels of myosin IIB are also seen during the stochastic ingression of presumptive mesendoderm cells in gastrulating mouse embryos. However, in this case the levels of myosin IIB correlate inversely with the ingressing cell, suggesting that these cells are extruded from the epiblast by neighbouring cells with high levels of apical myosin [11]. Interestingly, in Crumbs2 mutants where myosin IIB anisotropy is lost, basement membrane breakdown occurs, but the cells are stuck in the epiblast layer and do not undergo EMT, suggesting that basement membrane breakdown alone is not sufficient for EMT to occur. Taken together, developmental-EMTs suggest that EMT is achieved through the combined activation of multiple different cell behaviours, in a highly cell context dependent manner.

## Cadherin switching during developmental-EMTs

Cells undergoing EMT often display cadherin switching, where they downregulate one cadherin and induce expression of another, for example from E-Cadherin to N-Cadherin. This so-called ‘cadherin switch’ alters the cell–cell adhesion molecules relative to those of its tissue of origin and has been proposed to be required for a cell undergoing EMT to separate from its neighbours [37]. Interestingly, in Lamprey, a jawless vertebrate, neural crest migration is Snail-dependent, but has been shown to occur without a differential shift in cadherin expression, indicating that differential regulation of classical cadherin expression is not required to initiate neural crest migration in basal vertebrates [38]. Recent studies have investigated the functional requirement for cadherin switching during EMT in gastrulating *Drosophila* and chick embryos and *Xenopus* cranial neural crest cells, by modulating either E-Cadherin levels so that they cannot be switched off, or N-Cadherin, so that it cannot be switched on. These studies have elegantly proven that cadherin switching is not required for the segregation or dispersal of the mesodermal germ layer in *Drosophila* [26] or chicks [18^•^], nor in cranial neural crest cells in xenopus [23]. Thus, accumulating studies of developmental-EMTs suggest that a ‘cadherin switch’ is not required for cells undergoing EMT to separate from their tissue of origin.

Interestingly, an alternative functional role for E-cadherin to N-cadherin switch was recently identified during neural crest migration in *Xenopus* and zebrafish [27^••^]. After EMT and delamination from the neural tube, neural crest cells migrate extensively and differentiate into numerous cell lineages including melanocytes, neurons, glia, cartilage and bone. Central to neural crest cell migration is the ability of these cell to undergo contact inhibition of locomotion (CiL), whereby cells move away from each other after cell–cell contact [39,40]. When migrating cells contact each other, they initially downregulate their protrusions and form cell–cell contacts, and then generate a dominant lamellipodium away from the point of contact and detach. This causes mesenchymal cells to reorient their migration, typically moving in the direction away from their point of contact. By comparing premigratory and migratory neural crest cells, Scarpa *et al.* show that the switch from E-cadherin to N-cadherin is required for CiL. Overexpression of E-Cadherin in migratory neural crest cells impairs CiL through loss of protrusion formation, and cell–cell contacts are stabilised after collision. In contrast, when N-Cadherin alone is present, cell–cell contacts are only transiently formed, and traction forces driven by protrusion formation at the opposite edges are sufficient to pull the cells apart [27^••^,^41^]. These results are intriguing, as they suggest that migrating mesenchymal cells will respond differently to cell–cell contact, depending on the type of cadherin they express. Thus, mesenchymal cells which express E-Cadherin may form dynamic cell–cell contacts that favour a more cohesive migration such as in the *Drosophila* endoderm [14^••^], whereas cells with N-Cadherin will undergo CiL driving a more dispersed collective migration. However, it is likely that this is again highly cell type and context dependent.

## Cell-intrinsic and extrinsic influence of mechanical cues on the timing of an EMT

Accumulating studies suggest the EMT is highly cell-context dependent. Simply providing epithelial cells with cocktails of EMT-inducing signalling proteins does not necessarily result in induction of EMT in those cells. Developing tissues often express EMT-inducing transcription factors well before EMT takes place and recent studies have demonstrated an important role for mechanical cues in determining the timing of an EMT during normal development. For example, in *Drosophila,* the EMT-inducing transcription factor Snail is expressed in presumptive mesoderm cells well before EMT takes place. The timing of EMT is tightly controlled and only occurs after the mesoderm has been internalized [42]. A recent study showed that during invagination, increases in actomyosin contractility strengthens the junctions, and this overrides Snail-dependent junctional disassembly [33^••^]. EMT only occurs once cells are internalised and the actomyosin tension is released. Interestingly, ectopic expression of Snail in ectodermal epithelial cells was sufficient to drive EMT, but junctional disassembly is blocked by simultaneously inducing myosin contractility [33^••^]. Cell-extrinsic mechanical cues have also recently been implicated in influencing the timing of EMT. Neural crest cells express EMT-inducing transcription factors well in advance of the onset of migration [43^••^]. Expression of these transcription factors is not sufficient for EMT, an external trigger is required, which is provided by the stiffening of the underlying tissue, the head mesoderm. To detect changes in their mechanical environment, neural crest cells use mechanosensation mediated by the integrin-vinculin-talin complex [43^••^]. Taken together these studies suggest that cells integrate both molecular cues and tissue mechanics to coordinate EMT and tissue morphogenesis.

## Significance of emerging developmental-EMT concepts for the cancer field

Over the past years, the prevalent view in the cancer field has been that tumor cells undergo an EMT during the early stages of the metastatic cascade, increasing their motility and invasive capacities [44, 45, 46, 47, 48]. However, recent studies have suggested that EMT is not necessary for the generation of metastases [49,50], raising an intense debate on the importance of EMT in cancer [51, 52, 53, 54]. These studies lineage-traced a selection of mesenchymal markers in mouse models for metastatic cancer, in an attempt to track cancer cells that have undergone EMT activation [49,50]. They found that these markers were not expressed in metastases, leading the authors to suggest that cancer cells metastasise without activating EMT. An alternative view is that similar to many cells during development, cancer cells may only activate a partial-EMT en-route to forming metastases, and thus may not activate markers associated with a more extreme mesenchymal phenotype. In line with this, recent *in vivo* evidence has demonstrated the existence of multiple tumor subpopulations associated with many different EMT states, from epithelial to completely mesenchymal, passing through numerous intermediate hybrid states [55^••^]. Future studies using more sensitive and robust permanent tracing systems using markers derived from these intermediate hybrid states should help to illuminate the importance of EMT in cancer progression. Intriguingly, these intermediate states displayed differences in cellular plasticity, invasiveness and metastatic potential [55^••^]. It will be important in the future to understand how these differences in terms of markers relate back to cell morphological features, and the ability to migrate cohesively, as cell clusters are increasingly recognised as potent drivers of metastasis [56].

Recent studies also attempted to block EMT through the deletion of EMT-inducing transcription factors Snail1 and Twist1 or activation of miR-200, a putative suppressor of EMT. However, this failed to supress metastasis, suggesting that similar to developmental contexts, the roles of EMT-transcription factors in cancer cells are non-redundant as well as tissue-specific. For example, it was demonstrated that Snail triggers metastasis in breast cancer [57], whereas it has no effect on metastasis in a pancreatic cancer model [50]. However, in contrast to Snail, Zeb1 favours metastasis in pancreatic cancer [58]. Tumours are extremely heterogenous, and these emerging data demonstrate a context dependence to EMT in cancer cells, and suggest a huge diversity in underlying cellular mechanisms. A difficulty moving forward will be to identify definitive sets of markers for EMT, as it will likely require a different set for each tissue or even tumor type. Achieving a full-mechanistic understanding of EMT will be even more challenging, but as this diversity is also seen in embryos, cross-disciplinary studies drawing comparisons between developmental-EMTs and cancer-EMTs should drive this field forward.

## Conflict of interest statement

Nothing declared.

## References and recommended reading

Papers of particular interest, published within the period of review, have been highlighted as

• of special interest
•• of outstanding interest
